# Associations of A-FABP with Anthropometric and Metabolic Indices and Inflammatory Cytokines in Obese Patients with Newly Diagnosed Type 2 Diabetes

**DOI:** 10.1155/2016/9382092

**Published:** 2016-10-13

**Authors:** Guifen Niu, Jian Li, Huaiguo Wang, Yuan Ren, Jie Bai

**Affiliations:** ^1^Department of Endocrinology, People's Hospital of Liaocheng, Shandong 252000, China; ^2^Department of Urology, People's Hospital of Liaocheng, Shandong 252000, China

## Abstract

The study aimed to evaluate the relationship between anthropometric and metabolic indices, inflammatory cytokines, and adipocyte fatty acid-binding protein (A-FABP) in obese patients with newly diagnosed type 2 diabetes. The study included 48 nonobese subjects with newly diagnosed type 2 diabetes, 42 obese subjects with newly diagnosed type 2 diabetes, 30 simple obese subjects, and 30 matched normal subjects. Serum A-FABP was assessed by enzyme-linked immunosorbent assay. Pearson's correlations and multiple linear regression stepwise analysis were used to analyze correlations of A-FABP with anthropometric and metabolic indices and inflammatory cytokines. Obese subjects with newly diagnosed type 2 diabetes had elevated A-FABP compared to normal control, nondiabetic obese patients, and nonobese diabetic patients. A-FABP was significantly correlated with glycated hemoglobin A1C (HbA1C), BMI, triglyceride, Homeostasis Model Assessment Index (HOMA-IR), waist hip rate, C-reactive protein, IL-6, and HDL-C in obese subjects with type 2 diabetes. In multiple linear regression stepwise analysis, BMI, HbA1C, and HOMA-IR were significantly independent determinants for A-FABP. BMI, HbA1C, and HOMA-IR are independently associated with A-FABP in obese subjects with newly diagnosed type 2 diabetes. A-FABP may be related to insulin resistance and inflammation in type 2 diabetes and concomitant obesity.

## 1. Introduction

Diabetes is a chronic multisystem disease all over the world. Diabetes affects more than 180 million people all over the world, and the diabetes patients are anticipated to increase to 300 million by 2025 [1]. It has led to severe complications of peripheral nerves, cardiovascular system, eyes, and kidneys in an increasing number of patients [2, 3]. Besides, obesity is the determinant of type 2 diabetes, and diabetes often makes losing weight more difficult and brings about antidiabetic medications associated weight gain [4, 5]. These findings suggest that diabetes is closely associated with obesity. Diabetes and obesity have placed heavy health and economic burdens on people [2, 3].

Obesity is associated with adipose tissue inflammation and increased secretion of proinflammatory adipokines such as adipocyte fatty acid-binding protein (A-FABP), tumor necrosis factor-alpha (TNF-*α*), and interleukin-6 (IL-6) [6, 7]. A-FABP, also known as aP2 or FABP4, is a cytosolic lipid chaperone expressed primarily in activated macrophages and mature adipocytes [8]. It reversibly binds to hydrophobic ligands with high affinity [9]. Emerging evidence has suggested that A-FABP plays a vital role in lipid-mediated biological processes associated with type 2 diabetes, obesity, and metabolic syndrome [10, 11]. The inhibition of FABP4 can improve type 2 diabetes mellitus and atherosclerosis in mouse model, and elevated circulating FABP4 levels are associated with obesity, metabolic disease, and cardiac dysfunction in humans [12]. Even, higher plasma A-FABP levels increase cardiometabolic risk, and loss-of-function alleles in humans provide protection against cardiometabolic disease and mortality [13, 14]. Tuncman et al. indicate that humans with a functional genetic variant of the A-FABP gene have a significantly reduced risk for type 2 diabetes and coronary artery disease [15]. Moreover, serum A-FABP is associated with anthropometric parameters, atherogenic parameters, and insulin resistance in overweight/obese women [16]. Terra et al. has reported that serum FABP4 is positively correlated with TNF receptors, C-reactive protein (CRP), and IL-6 in morbidly obese women [17]. However, the relationships between anthropometric and metabolic indices, inflammatory cytokines, and A-FABP in obese patients with diabetes remain unclear.

In this study, inflammatory cytokines and anthropometric and metabolic indices were compared between healthy subjects, obese subjects without type 2 diabetes, nonobese subjects with type 2 diabetes, and obese subjects with type 2 diabetes. Moreover, the relationships of inflammatory cytokines and anthropometric and metabolic indices with A-FABP were also assessed.

## 2. Materials and Methods

### 2.1. Ethics Statement

The study was approved by the ethics committee of the People's Hospital of Liao Cheng. Written informed consent was obtained from all participants.

### 2.2. Participants

#### 2.2.1. Diabetes Group

A total of 90 outpatients and inpatients, who were newly diagnosed with type 2 diabetes according to the World Health Organization 1999 criteria [18] in endocrinology of the People's Hospital of Liao Cheng, were enrolled onto this study between March 2013 and October 2013. Exclusion criteria were type 1 diabetes, infection, hepatic disease, cardiovascular disease, acute or chronic inflammation, malignant tumor, and use of any medication known to affect bodyweight. These patients with newly diagnosed type 2 did not receive oral hypoglycemic agents or diet control. According to BMI (body mass index), type 2 diabetes patients were divided into nonobese (*n* = 48, BMI < 25 kg/m^2^) and obese (*n* = 42, BMI ≥ 25 kg/m^2^) groups.

#### 2.2.2. Simple Obese Group

According to the diagnostic criteria of obesity (BMI ≥ 25 kg/m^2^) by Chinese Medical Association Diabetes Branch, a group of 30 subjects with normoglycemia (<5.6 mmol/L; fasting glucose was measured twice within a week) were enrolled onto the study between March 2013 and October 2013. Exclusion criteria were acute illness; acute or chronic inflammatory or infective diseases; malignant disease; use of any medication known to affect bodyweight.

#### 2.2.3. Control Group

A comparable number of age-matched healthy subjects (BMI < 25 kg/m^2^) were also recruited and defined as the control group. These subjects did not have any personal or family history of diabetes, bone metabolic disease, liver and kidney dysfunction, history of tumor, cardiovascular and cerebrovascular diseases, asthma, or rheumatic disease.

All subjects in simple obese and control groups were screened by a physical examination in the examination center of hospital. Blood samples were drawn after an overnight fast and were kept at −80°C.

### 2.3. Anthropometric and Biochemical Measurements

All subjects were assessed after an overnight fast of at least 10 h. Anthropometric measurements including weight, height, and waist hip ratio (WHR) were measured by standard methods. BMI was calculated as weight/height (kg/m^2^). Biochemical variables were analyzed according to protocol of provider. The glucose oxidase method was applied to assess levels of fast blood glucose (FBG) and 2 h postprandial glucose (2hPG). Levels of serum lipid including total cholesterol (TC), triglyceride (TG), high-density lipoprotein cholesterol (HDL-C), low-density lipoprotein cholesterol (LDL-C), and very low-density lipoprotein cholesterol (VLDL-C) and parameters representing renal function including blood urea nitrogen (BUN), creatinine (Cr), and uric acid (UA) were detected using an enzymatic method (CX-7 biochemical autoanalyzer, Beckman Coulter, Inc., CA, USA). Glycated hemoglobin (HbA1C) was measured by high performance liquid chromatography (HPLC) using a variant hemoglobin HbA1C assay (D-10, Bio-Rad, Hercules, CA, USA). Levels of fasting insulin (FINS), C-peptide (C-P), and 2 h postprandial insulin (2hINS) and C-P (2hC-P) were measured using highly sensitive immunoenzymometric assay (IEMA) kits provided by Beijing Bio-EKon Biotechnology (Beijing, China). Intra- and interassay variation coefficients were 4% and 10%, respectively. These parameters were detected by ARIM-ZYME I tester (Aikang Biomedical Engineering Co., Ltd., Wuhan, Hubei province, China). Insulin resistance (IR), which was measured based on the Homeostasis Model Assessment Index (HOMA-IR), was calculated as FPG (mmol/L) × FINS (mU/L)/22.5 [19]. CRP, IL-6, interleukin-17 (IL-17), and A-FABP were measured using commercially available (enzyme-linked immunosorbent assay) ELISA kits purchased from Yes Biotech Laboratories Company (Toronto, Canada), Beijing 4A Biotech Company (Beijing, China), and Groundwork Biotechnology Diagnosticate Company (USA). Intra- and interassay variation coefficients were 5% and 10%, respectively.

### 2.4. Statistical Analysis

All data was analyzed using Statistical Package for the Social Sciences (SPSS Version 11.0, SPSS Inc., Chicago, USA). Data was presented as mean ± SD. Comparisons of continuous data between two groups were conducted using unpaired Student's *t*-test. Dichotomous variables between two groups were compared by chi-square test. Pearson correlations analysis was performed for normally distributed data to evaluate the association between A-FABP, anthropometric and metabolic indices, and inflammation factors. Multiple linear regression stepwise analysis was conducted to identify factors independently correlated with serum A-FABP. With A-FABP as dependent variable, the significant factors derived from Pearson's correlations analysis were considered as independent variables for the regression model. Only the variables with *P* < 0.05 were included in the final fitted model. A *P* value < 0.05 was set as the cutoff for statistical significance.

## 3. Results

### 3.1. Baseline Characteristics and Anthropometric Indices of Subjects

Baseline characteristics of all study subjects (*n* = 150) in normal control (*n* = 30), simple obesity (*n* = 30), type 2 diabetes without obesity (*n* = 48), and type 2 diabetes with obesity (*n* = 42) groups were presented in Table 1. Age and gender distribution did not differ significantly among the four groups. The subjects in the simple obese group and the type 2 diabetes with diabetes group had significantly higher BMI and WHR compared with the normal control group (*P* < 0.05). Additionally, the type 2 diabetes without obesity group had lower BMI and WHR than the simple obesity group (*P* < 0.05). Moreover, BMI and WHR were significantly elevated in the type 2 diabetes with obesity group compared to those in the type 2 diabetes without obesity group (*P* < 0.05).

### 3.2. Comparison of Metabolic Parameters among Groups

Metabolic parameters of different groups were shown in Table 2. Simple obesity, type 2 diabetes without obesity, and type 2 diabetes with obesity groups displayed significantly higher levels of TC and TG and lower level of HDL-C compared with the control group (*P* < 0.05). Among the three groups, type 2 diabetes with obesity group had significantly elevated TG and LDL-C and decreased HDL-C in comparison with the simple obese group and the type 2 diabetes without obesity group (*P* < 0.01). Additionally, TG, LDL-C, and HDL-C were not significantly different between the simple obese group and the type 2 diabetes without obesity group (*P* > 0.05). Besides, VLDL-C in type 2 diabetes with or without obesity groups was significantly higher than that in normal control and simple obesity groups (*P* < 0.05). The difference in VLDL-C between type 2 diabetes with obesity group and type 2 diabetes without obesity group was not significantly different (*P* > 0.05).

Additionally, renal function-related parameters BUN, Cr, and UA were not significantly different between the 4 groups (*P* > 0.05, Table 2). For the serum glucose biomarkers (Table 3), type 2 diabetes with or without obesity groups had markedly higher levels of FBG, 2hPG, HbA1C, FINS, 2hINS, 0hC-P, 2hC-P, and HOMA-IR relative to the normal control or simple obesity group (*P* < 0.05). Moreover, the type 2 diabetes with obesity group had increased HOMA-IR compared to the type 2 diabetes without obesity group (*P* < 0.05).

### 3.3. Comparison of A-FABP and Inflammatory Cytokines among Groups

The levels of CRP, IL-6, IL-17, and A-FABP in simple obesity and diabetes with or without obesity groups were obviously increased compared to those in the normal control group (*P* < 0.05, Table 4). Among the 3 groups, the diabetes with or without obesity groups had increased levels of CRP, IL-6, IL-17, and A-FABP compared to the simple obesity group (*P* < 0.05). Furthermore, levels of IL-6 and A-FABP were significantly higher in the diabetes with obesity group compared with those in the diabetes without obesity group (*P* < 0.05, Table 4).

### 3.4. Pearson's Correlations Analysis and Multiple Stepwise Regression Analysis

Pearson's correlations analysis was performed to identify factors correlated with A-FABP in obese patients with type 2 diabetes (Table 5). After adjustment for age and sex, HbA1C (*r* = 0.56, *P* < 0.01), BMI (*r* = 0.45, *P* < 0.01), TG (*r* = 0.41, *P* < 0.01), HOMA-IR (*r* = 0.36, *P* < 0.05), WHR (*r* = 0.34, *P* < 0.05), CRP (*r* = 0.2, *P* < 0.05), and IL-6 (*r* = 0.2, *P* < 0.05) positively correlated with A-FABP (Table 5). The only parameter that negatively correlated with A-FABP was HDL-C (*r* = −0.22, *P* < 0.05). FBG, 2hPG, TC, LDL-C, VLDL-C, BUN, Cr, UA, FINS, 2h-INS, 0hC-P, 2hC-P, and IL-17 did not significantly correlate with A-FABP (*P* > 0.05). Furthermore, the multiple stepwise regression analysis unveiled that BMI, HbA1C, and HOMA-IR were independent determinants of A-FABP (*P* < 0.05). The regression equation was *Y*_A-FABP_ = 0.512*X*_BMI_ + 6.482_HbA1C_ + 1.21_HOMA-IR_ − 4.31.

## 4. Discussion

This study found elevated A-FABP level in subjects with newly diagnosed type 2 diabetes compared with normal control or simple obese subjects. The obese subjects with newly diagnosed type 2 diabetes had further increased A-FABP level compared to the nonobese subjects with newly diagnosed type 2 diabetes. Moreover, A-FABP was found to be associated with several variables including HbA1C, BMI, TG, HDL-C, HOMA-IR, WHR, CRP, and IL-6 in obese subjects with type 2 diabetes. Of these variables, BMI, HbA1C, and HOMA-IR were significantly independent determinants for A-FABP.

FABPs are a family of proteins that participate in shuttling fatty acids to cellular compartments, modulating intracellular lipid metabolism and regulating gene expression [20]. A previous study has found obviously increased level of A-FABP in obese children compared with normal-weight children [21]. Similarly, higher level of A-FABP has been observed in Korean children with greater BMI, WHR, higher levels of HOMA-IR and TAG, and lower level of HDL-C [22]. This study confirmed the increase of A-FABP in obese subjects relative to nonobese subjects. Moreover, the subjects with impaired glucose tolerance or fasting glucose possess higher levels of A-FABP in Chinese population, and serum A-FABP predicts the development of type 2 diabetes [11]. This study also provided evidence that A-FABP was elevated in subjects with newly diagnosed type 2 diabetes compared to normal controls. Furuhashi et al. have found that targeting A-FABP with small-molecule inhibitor can prevent and treat type 2 diabetes and atherosclerosis [23]. Thus, A-FABP may be a promising target for obese patients with type 2 diabetes. More studies are needed to elucidate the role of A-FABP in type 2 diabetes and concomitant obesity.

Considerable emphasis has been placed on the relationship of A-FABP with anthropometric indices and factors associated with insulin resistance, atherogenesis, and obesity. One instance is a previous study showing that plasma A-FABP in morbidly obese women is positively correlated with BMI and HOMA-IR [17]. Besides, evidence suggests that serum A-FABP is associated with insulin resistance and anthropometric and atherogenic indices in young overweight and obese women [16]. Moreover, increasing studies have revealed positive relations between BMI, WHR, HOMA-IR, FINS, TG, and A-FABP and negative relation between HDL-C and A-FABP in type 2 diabetic patients [22, 24]. However, the relationships of A-FABP with anthropometric indices and the factors associated with insulin resistance and atherogenesis in obese subjects with newly diagnosed type 2 diabetes remain elusive. The results of the current study showed that HbA1C, BMI, TG, HOMA-IR, WHR, CRP, and IL-6 were positively correlated with serum A-FABP, and HDL-C was negatively correlated with A-FABP in obese subjects with newly diagnosed type 2 diabetes. Nonetheless, FINS was not significantly correlated with A-FABP in the present study. The reason may be that the number of patients was small. Multiple stepwise regression analysis further proved that BMI, HbA1C, and HOMA-IR were significantly independent determinants for A-FABP. It suggested that A-FABP might be associated with insulin resistance in obese patients with newly diagnosed type 2 diabetes. In line with our present findings, Roden reported that increased circulating A-FABP level was associated with insulin resistance and type 2 diabetes [25]. Furthermore, Mölig et al. reported that the level of circulating A-FABP was an obesity associated marker [26].

Chronic inflammation is considered to be associated with obesity and insulin resistance [27]. A-FABP participates in regulating the production of inflammatory cytokines [28]. Positive association of serum FABP4 with inflammation factors TNF receptors, CRP, and IL-6 has been observed in morbidly obese women [17]. Terra et al. found that CRP, IL-6, and IL-17 were significantly higher in patients with newly type 2 diabetes compared to those in nondiabetic obese patients and normal healthy subjects, and A-FABP was positively correlated with CRP and IL-6 in obese subjects with newly diagnosed type 2 diabetes [17]. It suggested that A-FABP might be associated with inflammation in type 2 diabetes and concomitant obesity. In the current study, there was no significant correlation between increased IL-17 and A-FABP in obese subjects with type 2 diabetes. One study shows that IL-17 promotes inflammation through effects of receptors that trigger downstream cytokine production [29]. Previous report indicates that IL-17 immunity plays a vital role in human type 1 diabetes and IL-17 may be a potential therapeutic target [30]. Additionally, IL-17 production is associated with severity of type 2 diabetes [31]. However, the role of IL-17 in type 2 diabetes has to be further determined. More inflammatory cytokines such as TNF-*α* and TNF receptors will be tested in further studies.

## 5. Conclusions

In summary, the study suggests that BMI, HbA1C, and HOMA-IR are associated with serum A-FABP in obese subjects with newly diagnosed type 2 diabetes. A-FABP may be related to insulin resistance and inflammation in type 2 diabetes and concomitant obesity. Further studies with a large number of patients are needed to verify the findings of the study.

## Figures and Tables

**Table 1:** Baseline clinical characteristics of subjects in different groups.

Group	Subjects	Male/female	Age	BMI (kg/m^2^)	WHR
Normal control	30	16/14	50 ± 4.8	22.42 ± 1.3	0.81 ± 0.03
Simple obesity	30	14/16	49 ± 7.6	28.76 ± 1.8^☆^	0.87 ± 0.06^☆^
Type 2 diabetes					
Nonobese	48	23/25	49 ± 5.6	23.04 ± 1.2^○^	0.82 ± 0.04^○^
Obese	42	22/20	50 ± 7.8	29.11 ± 1.6^☆∆^	0.88 ± 0.05^☆∆^

Data are mean ± SD or median. BMI: body mass index; WHR: waist hip rate. ^☆^ *P* < 0.05 versus normal control; ^○^ *P* < 0.05 versus simple obese group; ^∆^ *P* < 0.05 versus nonobese diabetic group.

**Table 2:** Metabolic parameters of subjects in different groups.

Group	TC (mmol/L)	TG (mmol/L)	HDL-C (mmol/L)	LDL -C (mmol/L)	VLDL-C (mmol/L)	BUN (mmol/L)	Cr (umol/L)	UA (umol/L)
Normal control	3.62 ± 0.76	1.26 ± 0.53	1.44 ± 0.23	2.42 ± 0.83	0.52 ± 0.07	4.20 ± 1.7	42.0 ± 5.7	204 ± 17
Simple obesity	4.67 ± 0.58^☆^	1.56 ± 0.62^☆^	1.35 ± 0.37^☆^	2.46 ± 0.72	0.51 ± 0.03	3.96 ± 1.5	43.0 ± 3.2	212 ± 12
Type 2 diabetes								
Nonobese	4.65 ± 0.61^☆^	1.60 ± 0.66^☆☆^	1.27 ± 0.35^☆☆^	2.52 ± 0.57	0.71 ± 0.04^☆○^	4.32 ± 0.8	46.0 ± 4.2	207 ± 16
Obese	5.21 ± 0.72^☆☆^	2.31 ± 1.27^☆☆○○∆∆^	0.95 ± 0.42^☆☆○○∆∆^	2.83 ± 0.64^☆☆○○∆∆^	0.74 ± 0.06^☆○^	4.48 ± 0.6	43.0 ± 5.0	215 ± 27

TC: total cholesterol; TG: triglyceride; HDL-C: high-density lipoprotein cholesterol; LDL-C: low-density lipoprotein cholesterol; VLDL-C: very low-density lipoprotein; BUN: blood urea nitrogen; Cr: creatinine; UA: uric acid. Data are mean ± SD or median. ^☆^ *P* < 0.05, ^☆☆^ *P* < 0.01 versus normal control; ^○^ *P* < 0.05, ^○○^ *P* < 0.01 versus simple obese group; ^∆∆^ *P* < 0.01 versus nonobese diabetic group.

**Table 3:** Comparisons of blood biomarkers of study subjects.

Group	FBG (mmol/L)	2hPG (mmol/L)	HbA1C (%)	FINS (mu/L)	2hINS (mu/L)	C-P (ng/mL)	2hC-P (ng/mL)	HOMA-IR
Normal control	4.71 ± 1.2	5.72 ± 1.8	4.7 ± 0.4	13.7 ± 3.6	43.2 ± 7.63	1.46 ± 0.78	4.32 ± 1.30	2.91 ± 1.10
Simple obesity	4.96 ± 1.06	5.96 ± 1.23	5.1 ± 0.3	17.2 ± 1.7^☆^	40.1 ± 3.72	1.56 ± 0.62	4.30 ± 0.98	4.70 ± 1.24^☆^
Type 2 diabetes								
Nonobese	9.25 ± 3.12^☆☆○○^	12.56 ± 1.03^☆☆○○^	8.7 ± 1.2^☆☆○○^	23.6 ± 4.7^☆☆^	52.7 ± 4.6^☆☆○○^	3.03 ± 0.32^☆○^	7.98 ± 0.72^☆☆○○^	8.47 ± 3.7^☆☆○○^
Obese	9.65 ± 2.16^☆☆○○^	13.76 ± 2.53^☆☆○○^	8.9 ± 0.9^☆☆○○^	25.2 ± 8.7^☆☆^	49.3 ± 5.3^☆☆○○^	3.06 ± 0.43^☆○^	8.76 ± 0.69^☆☆○○^	11.3 ± 2.6^☆☆○○∆^

FBG: fasting plasma glucose; 2hPG: 2 h postchallenge plasma glucose; HbA1C: glycated hemoglobin A1C; FINS: fasting insulin; 2hINS: 2 h postprandial insulin; C-P: C-peptide; 2hC-P: 2 h postprandial C-peptide; HOMA-IR: Homeostasis Model Assessment Index of insulin resistance. Data are mean ± SD or median. ^☆^ *P* < 0.05, ^☆☆^ *P* < 0.01 versus normal control; ^○^ *P* < 0.05, ^○○^ *P* < 0.01 versus simple obese group; ^∆^ *P* < 0.05 versus nonobese diabetic group.

**Table 4:** Comparisons of serum cytokines and A-FABP in study subjects.

Group	CRP (mg/L)	IL-6 (pg/mL)	IL-17 (pg/mL)	A-FABP (ng/mL)
Normal control	1.7 ± 0.8	106.7 ± 7.5	17.5 ± 1.2	4.37 ± 2.56
Simple obesity	2.8 ± 0.3^☆^	126.9 ± 6.4^☆^	23.2 ± 0.6^☆^	10.32 ± 2.75^☆^
Type 2 diabetes				
Nonobese	4.3 ± 0.5^☆☆○○^	260 ± 18.4^☆☆○○^	36.8 ± 4.2^☆☆○○^	16.74 ± 2.13^☆☆○^
Obese	4.7 ± 0.3^☆☆○○^	344 ± 17.9^☆☆○○∆∆^	34.5 ± 6.8^☆☆○○^	21.38 ± 2.12^☆☆○○∆^

CRP: C-reactive protein; IL-6: interleukin-6; IL-17: interleukin-17; A-FABP: adipocyte fatty acid-binding protein. Data are mean ± SD or median. ^☆^ *P* < 0.05, ^☆☆^ *P* < 0.01 versus normal control; ^○^ *P* < 0.05, ^○○^ *P* < 0.01 versus simple obese group; ^∆^ *P* < 0.05, ^∆∆^ *P* < 0.01 versus nonobese diabetic group.

**Table 5:** Identification of the factors correlated with A-FABP in obese patients with type 2 diabetes.

	IL-6	CRP	IL-17	A-FABP	BMI	WHR	HOMA-IR
IL-6	—						
CRP	0.15	—					
IL-17	0.08	0.03	—				
A-FABP	0.20^☆^	0.20^☆^	0.11	—			
BMI	0.17	0.20^☆^	0.06	0.45^☆☆^	—		
WHR	0.16	0.19	0.08	0.34^☆^	0.45^☆☆^	—	
TG	0.20^☆^	0.22^☆^	0.04	0.41^☆☆^	0.42^☆☆^	0.36^☆☆^	
HDL-C	−0.20^☆^	−0.21^☆^	−0.13	−0.22^☆^	−0.25^☆☆^	−0.23^☆^	
HbA1C	0.252^☆☆^	0.23^☆^	0.15	0.56^☆☆^	0.27^☆☆^	0.20^☆^	
HOMA-IR	0.26^☆☆^	0.21^☆^	0.14	0.36^☆^	0.30^☆☆^	0.22^☆^	—

IL-6: interleukin-6; CRP: C-reactive protein; IL-17: interleukin-17; A-FABP: adipocyte fatty acid-binding protein; BMI: body mass index; WHR: waist hip rate; TG: triglyceride; HDL-C: high-density lipoprotein cholesterol; HbA1C: glycated hemoglobin A1C; HOMA-IR: Homeostasis Model Assessment Index of insulin resistance. ^☆^ *P* < 0.05, ^☆☆^ *P* < 0.01.
